# Attachment-Based Support and Depression in Adult Child-Parent Dyads: The Role of Secure Base and Safe Haven

**DOI:** 10.1093/geroni/igaf122.1749

**Published:** 2025-12-31

**Authors:** Ella Carasso, Dikla Segel-Karpas, Shira Barzilay, Roi Estlein

**Affiliations:** University of Haifa, Cambridge, Massachusetts, United States; University of Haifa, Haifa, HaZafon, Israel; University of Haifa, Haifa, HaZafon, Israel; University of Haifa, Haifa, HaZafon, Israel

## Abstract

The adult child-parent relationship is a key source of mutual support, promoting psychological well-being in both generations. This study examined the dynamics of support between adult children and their parents through an attachment framework, focusing on how two forms of attachment-based social support, secure base and safe haven, are associated with depressive symptoms in both generations. A total of 129 adult child-parent dyads (parents’ age: M = 69.63; adult children’s age: M = 42.02) participated. Multilevel modeling (MLM) was used to assess associations between depressive symptoms and attachment-based support dynamics. Results showed that parents’ provision of a safe haven and secure base was negatively associated with their own depressive symptoms, but not with their children’s. Adult children’s provision of a secure base, but not a safe haven, was negatively associated with their own depressive symptoms. These findings suggest that providing a secure base and safe haven may help parents preserve their parental role, enhancing resilience in older age and mitigating depression. Moreover, adult children’s provision of a secure base to aging parents may support their mental health by balancing the support of their parents’ autonomy with their own life goals.

